# Giant Enhancement of Exchange Coupling in Entropy-Stabilized Oxide Heterostructures

**DOI:** 10.1038/s41598-017-13810-5

**Published:** 2017-10-17

**Authors:** P. B. Meisenheimer, T. J. Kratofil, J. T. Heron

**Affiliations:** University of Michigan, Department of Materials Science and Engineering 2300 Hayward St, Ann Arbor, 48109 USA

## Abstract

Entropy-stabilized materials are stabilized by the configurational entropy of the constituents, rather than the enthalpy of formation of the compound. A unique benefit to entropy-stabilized materials is the increased solubility of elements, which opens a broad compositional space, with subsequent local chemical and structural disorder resulting from different atomic sizes and preferred coordinations of the constituents. Known entropy-stabilized oxides contain magnetically interesting constituents, however, the magnetic properties of the multi-component oxide have yet to be investigated. Here we examine the role of disorder and composition on the exchange anisotropy of permalloy/(Mg_0.25(1-x)_Co_x_Ni_0.25(1-x)_Cu_0.25(1-x)_Zn_0.25(1-x)_)O heterostructures. Anisotropic magnetic exchange and the presence of a critical blocking temperature indicates that the magnetic order of the entropy-stabilized oxides considered here is antiferromagnetic. Changing the composition of the oxide tunes the disorder, exchange field and magnetic anisotropy. Here, we exploit this tunability to enhance the strength of the exchange field by a factor of 10x at low temperatures, when compared to a permalloy/CoO heterostructure. Significant deviations from the rule of mixtures are observed in the structural and magnetic parameters, indicating that the crystal is dominated by configurational entropy. Our results reveal that the unique characteristics of entropy-stabilized materials can be utilized and tailored to engineer magnetic functional phenomena in oxide thin films.

## Introduction

In an entropy-stabilized material, the configurational entropic contribution (${\rm{\Delta }}{S}_{conf}$) to the Gibbs’ free energy, given by ${\rm{\Delta }}G={\rm{\Delta }}H-T{\rm{\Delta }}{S}_{conf}$, drives the formation of a single phase solid solution^1,2^. These materials have attracted significant interest due to the apparent deviations from Gibbs phase rule and desirable properties such as increased hardness, toughness, and corrosion resistance^1,3–6^. In particular, high entropy materials have been targeted for use in extreme temperature applications, as entropy domination prevents phase segregation and inhibits defect formation at high temperatures^7–9^. Despite the discovery of high entropy crystals nearly 15 years ago, reported investigations outside of transition metal alloys are limited^10–12^. Recent work^10^ has shown that the concept can be extended to cationic configurational disorder in binary oxides, where solid solution behavior is observed across the cation sites. As the magnetic and electronic properties of oxides are strongly correlated to their chemistry and electronic structure^13–15^, the increased solubility of species and disorder inherent to entropy stabilization could lead to exotic and colossal functional properties. For instance, recent reports of a colossal dielectric constant^16^ and superionic conductivity^17^ have been made in entropy-stabilized oxides. Here, we propose to take advantage of the inherent chemical and structural disorder in entropy stabilized oxides to enhance the exchange bias in ferromagnetic/antiferromagnetic heterostructures.

Due to the chemical disorder, entropy-stabilized oxides provide a system for investigating the contribution of configurational entropy to magnetic structure and interface exchange. The exchange bias effect is thought to be driven by frustrated or uncompensated spins near the ferromagnet (FM)/antiferromagnet (AFM) interface^18,19^, thus the local chemical disorder inherent to high entropy materials may result in a large increase of the interface exchange coupling through frustrated superexchange and uncompensated spin creation via the incorporation of non-magnetic species^20–23^. In the parent composition, rocksalt (Mg_0.2_Co_0.2_Ni_0.2_Cu_0.2_Zn_0.2_)O, three of the five binary oxide constituents are antiferromagnetic, with Néel temperatures of 289 K, 523 K, and 230 K for CoO (rocksalt), NiO (rocksalt), and CuO (tenorite) respectively. Additionally, two of the species, Cu and Zn, prefer tetrahedral coordination which may lead to a large degree of structural disorder in the material^24–26^. Considering these observations, we present a study of FM/(Mg_0.25(1-x)_Co_x_Ni_0.25(1-x)_Cu_0.25(1-x)_Zn_0.25(1-x)_)O exchange bias thin film heterostructures that reveals (Mg_0.25(1-x)_Co_x_Ni_0.25(1-x)_Cu_0.25(1-x)_Zn_0.25(1-x)_)O films are antiferromagnetic with properties which are strongly dependent on Co concentration, and by extension the degree of disorder. Due to composition driven changes to the magnetic structure, the exchange field can be increased by a factor of 10x at low temperatures^18,27,28^, when compared to a permalloy/CoO heterostructure.

## Results

### Pulsed Laser Deposition

**Figure 1:**
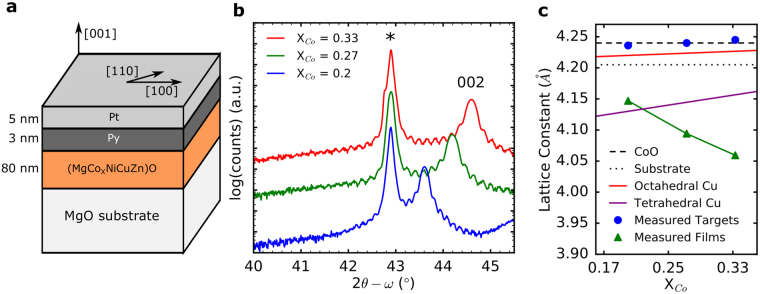
(**a**) Diagram showing the exchange bias heterostructure grown by pulsed laser deposition. (Mg_0.25(1-x)_Co_x_Ni_0.25(1-x)_Cu_0.25(1-x)_Zn_0.25(1-x)_)O is denoted as (MgCo_x_NiCuZn)O in the schematic. (**b)** 2*θ*-*ω* X-ray diffraction (XRD) data showing the substrate peak at 42.9°, marked with *, and film 002 peaks at 43.6°, 44.19°, and 44.59° for X = 0.20, 0.27, and 0.33, respectively. The films are single crystal and epitaxial with out-of-plane lattice constants that are dependent on Co concentration. The Laue oscillations about the film peaks show that the interfaces are flat and agree with our expected thicknesses of approximately 80 nm. The peak at ~46° in the X = 0.20 sample belongs to Pt 002 and results from a slightly thicker capping layer on the structure (7 nm, as opposed to 4 nm). (**c**) Lattice constant derived from part (**b**) and from the bulk (Targets) materials (Sup. Figure 5) plotted as a function of increasing Co concentration. Lattice constants were determined using Cohen’s method^46^ with calculated uncertainty smaller than the marker (Sup. Figure 2). The films display an opposite trend than would be expected from Vegard’s law using the ionic radii values of either octahedrally (red) or tetrahedrally (purple) coordinated Cu^2+^ and Zn^2+^, where the lattice constant should linearly approach that of CoO (dashed line). Interestingly, bulk values are in fair agreement with Vegard’s law with all cations octahedrally coordinated. The deviation may be due to thermodynamic defects as a result of high temperature quenching.

Thin films of (Mg_0.2(1-x)_Co_x_Ni_0.2(1-x)_Cu_0.2(1-x)_Zn_0.2(1-x)_)O (X = 0.2, 0.27, 0.33), hereafter denoted X = 0.20, X = 0.27, and X = 0.33, were grown by pulsed laser deposition on single crystal MgO (001) substrates and the nominal composition was confirmed using X-ray photoelectron spectroscopy (XPS) (Sup. Figure 1). A varying composition of Co was chosen to evaluate the effect of entropy and magnetism in these samples because CoO is a well-studied AFM that has a Néel temperature of 289 K, conveniently near room temperature and accessible for our measurements. Above 0.33 mole fraction Co in the samples, the growth conditions begin to drift and the films are no longer of comparable quality and thus not studied here (X = 1 = CoO being the exception). A schematic of the structure is shown in Fig. 1a. This geometry was chosen to minimize thickness effects in the AFM and to maximize exchange coupling^29^.

### X-ray Diffraction

2*θ*-*ω* scans, Fig. 1b, show that our films are single phase, single crystalline, and epitaxial to the (001)-oriented MgO substrate. The full spectrum diffraction pattern is provided in Sup. Figure 2. The Laue oscillations about the film peaks in Fig. 1b show that the film surfaces are smooth and atomic force microscopy of bare films (Sup. Figure 3) agrees, showing a root-mean-squared roughness of approximately 0.250 nm for each film. The period of the Laue oscillations about the 002 diffraction peak (Fig. 1b) and X-ray reflectometry (Sup. Figure 4) agree with our expected thickness of 75–80 nm. As we increase the mole fraction of Co in our oxide films, an approximately linear decrease in the out-of-plane lattice constant is observed, shown in Fig. 1c, while the in-plane lattice constant remains approximately constant due to epitaxial clamping from the substrate (Sup. Figure 4). Curiously, this is the opposite trend from what would be predicted using Vegard’s law, using a weighted average of the ionic radii of the constituent species. For this calculation, all cations were assumed to be in a 2 + oxidation state based on XPS spectra (Sup. Figure 1) and either all octahedrally (red) or with Cu^2+^ and Zn^2+^ tetrahedrally (purple) coordinated^30^. Interestingly, the bulk lattice parameter (blue points) is in fair agreement with Vegard’s law with all cations octahedrally coordinated. It has been observed in entropy-stabilized oxides that the lattice is locally distorted due to the presence of species that tend to Jahn-Teller distort, specifically Cu^2+^, which prefers a tetrahedral coordination to break orbital degeneracy^25^. Tetrahedrally coordinated Cu^2+^ and Zn^2+^ are much smaller (0.71 Å and 0.74 Å respectively^30^) than octahedral Cu^2+^ and Zn^2+^ (0.87 Å and 0.88 Å respectively^30^), potentially leading to a much smaller expected lattice constant. This does not, however, explain the negative trend with increasing Co incorporation seen in the lattice constant. This trend is also opposite to what would be expected from strain effects: as Co is the largest of the constituent cations^30^, increasing the concentration of Co should create a larger compressive strain in-plane due to clamping and the film would be expected to expand out-of-plane in accordance with Hooke’s law^31^. The contraction observed in the films is very large, corresponding to a strain of −2.2%, −3.6%, and −4.5% with respect to the bulk for X = 0.20, X = 0.27, and X = 0.33 respectively (Sup. Figure 6). If the observed trend in the lattice constant cannot be explained by potential changes in chemistry or epitaxial strain, we assert that it may be due to the structural distortions caused by the preferred coordination of the Cu atoms locally expanding the lattice. If this were the case, as a similar effect has been observed in some Perovskite systems^32,33^, reduction in the concentration of Cu could be expected to shrink the lattice constant faster than the increase due to higher concentration of larger (0.885 Å) Co^2+^ atoms^30^. As the strain values observed from XRD are large, we expect physical distortion from the ideal rock salt structure is large and thus there is a significant corresponding influence on the distortion driven interface exchange coupling^34–36^.

### Exchange Bias

**Figure 2:**
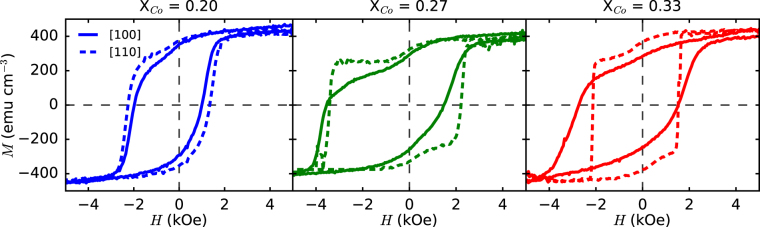
Plots of magnetic hysteresis at 10 K showing the exchange bias of the entropy-stabilized oxide exchange bias heterostructures containing varying compositions of Co. An increase in the concentration of Co changes the magnitude of the bias field and the anisotropy. The [110] easy axis anisotropy increases with molar fraction of Co.

A bias field and coercive field enhancement are the hallmarks of exchange bias behavior in FM/AFM bilayers. To explore the possibility of exchange bias in FM/entropy-stabilized oxide bilayers, field-dependent magnetometry measurements were made on the Pt/Py/(Mg_0.2(1-x)_Co_x_Ni_0.2(1-x)_Cu_0.2(1-x)_Zn_0.2(1-x)_)O samples presented in Fig. 1. Upon cooling the samples from 350 K to 10 K in a 2 T magnetic field, a significant exchange bias (0.5–1 kOe) and coercive field (1.5–2.5 kOe) are observed along [100] and [110] crystallographic directions at 10 K (Fig. 2). Consistent with exchange bias bilayers, the bias field changes sign with reversed cooling field polarity (Sup. Figure 7) and the coercive field is enhanced with respect to that of a Pt/Py/MgO control sample at 10 K (0.14 kOe). Magnetic anisotropy is present in all cases with the [110] axis being the easier axis. This becomes more pronounced as the concentration of Co is increased. The ratio of the relaxation energies between the [110] and [100] crystallographic directions, $\frac{{K}_{110}}{{K}_{100}}$, changes from 0.26, to 0.23, to 0.12 for X = 0.20, 0.27, and 0.33 respectively (Sup. Figure 8), revealing that [110] becomes the more favorable axis as Co concentration increases. For the X = 0.27 and X = 0.33 samples, the loops along the [110] axis become sharp indicating a [110] easy axis. The observed anisotropy indicates the presence of long-range magnetic order within the entropy-stabilized oxide and, with exchange bias, is suggestive of antiferromagnetism.

**Figure 3:**
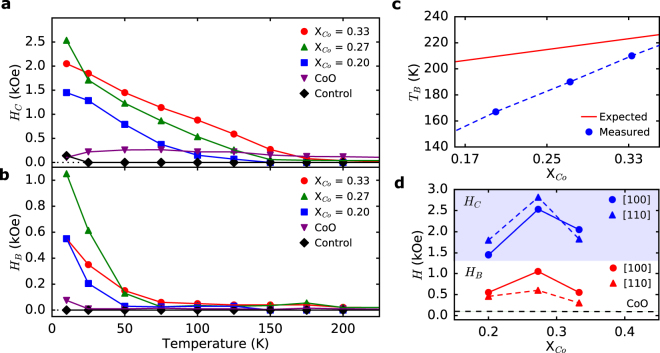
Plot of (**a**) coercive field and (**b**) exchange bias field, measured along the [100] crystallographic axis, versus temperature for entropy-stabilized oxide exchange bias heterostructures containing varying compositions of Co, plotted with a Pt/Py/CoO/MgO heterostructure (labeled CoO in the figure) and Pt/Py/MgO control sample. (**c**) The blocking temperatures (*T*_*B*_) extracted from Sup. Figure 9 (Measured), along with our prediction using the rule of mixtures (Expected), obtained by taking a weighted average of the individual Néel temperatures of the constituent binary oxides. The blocking temperature deviates significantly from the rule of mixtures, but approaches the expected value as the concentration is increased. The dashed line is provided as a guide to the eye. (**d**) Plot showing the change in coercive (*H*_*C*_) and bias (*H*_*B*_) fields as a function of Co concentration along both the [100] and [110] crystallographic directions at 10 K. The dashed line at 0.075 kOe corresponds to the bias field in the Pt/Py/CoO/MgO control sample. Error bars are smaller than the points for all plots.

However, in exchange bias FM/AFM systems, the coercivity enhancement and exchange bias must vanish at the blocking or Néel temperature. Figures 3a and 3b show temperature dependent exchange bias and coercive fields, along with those obtained from Pt/Py/CoO/MgO and Pt/Py/MgO reference samples, extracted from isothermal hysteresis loops taken every 25 K. Above ~200 K, the bias field and enhanced coercivity (with respect to a Pt/Py/MgO control sample) vanish for the entropy-stabilized oxide bilayer samples. For a more accurate determination of the blocking temperature (*T*_*B*_) as a function of Co concentration, field cooled (FC) and zero field cooled (ZFC) moment versus temperature curves (Sup. Figure 9) were measured. *T*_*B*_ was determined by the intersection of the FC and ZFC curves and reveals the onset temperature of the interaction between the two magnetic layers. A linear increase in *T*_*B*_ is observed (Fig. 3b) with increasing Co incorporation. The vanishing of the exchange bias field and coercive field enhancement above the blocking temperature, combined with the observation that films with no Py layer show no measurable magnetic moment, indicates that these entropy-stabilized oxides are antiferromagnetic. The results of Figs 2 and 3 also show that as the concentration of Co is increased, the entropy-stabilized crystal can be engineered to take on properties of the constituents, such as increased anisotropy and blocking temperature^22,23,37^, while still utilizing the increased disorder inherent to the system.

## Discussion

In all our studied entropy-stabilized oxides, the exchange bias is significantly greater than what is observed in the Pt/Py/CoO/MgO heterostructure (Fig. 3a,b). Our measured values for the Pt/Py/CoO/MgO sample agree with published results^18,27^, in which it was concluded that the spins on the (001) interface of CoO are well compensated and thus result in a small exchange bias (~0.1 kOe), even at low temperatures. Since it is known that there is significant local structural and chemical disorder in entropy-stabilized oxides^24^, it would be expected that the magnetic lattice becomes frustrated on a local scale. This could result in a significant increase in the exchange strength, as it has been observed that exchange bias is strongly influenced by the density of disordered magnetic moments at the FM/AFM interface^18,19,38–40^. We also suspect that this is the cause of the anomalous shape in the hysteresis curves shown in Fig. 2. According to the domain state model for exchange bias, a strong interfacial coupling between the FM and AFM can result in a large number of uncompensated moments at the interface which show hysteretic behavior in a shape similar to that of our experiments^41^. Additionally, in accordance with the domain state model, chemical dilution is predicted, and experimentally seen, to increase exchange bias due to the reduced energy cost for domain formation on impurity sites in the antiferromagnet^20,42^.

The blocking temperature is expected to be dominated by the number (relative total fraction) of magnetic ions and the strength of the superexchange interaction between them. This trend is expected and agrees, in terms of direction, with an estimation using the rule of mixtures from the Néel temperatures of the constituent oxides, which assumes that the Cu^2+^ sites will tend to distort and impart properties similar to those of the tenorite phase (*T*_*N*_ = 230 K) instead of a possible rocksalt phase, which may have a higher Néel temperature based on the trend shown by MnO (*T*_*N*_ = 122 K), FeO (*T*_*N*_ = 198 K), CoO (*T*_*N*_ = 289 K), and NiO (*T*_*N*_ = 523 K) rocksalts^43,44^. The measured *T*_*B*_, however, is lower than would be predicted and could be due to the local distortions inherent to the material which break local symmetry and could frustrate superexchange interactions. A potentially higher T_N_ from Cu^2+^ in an octahedral coordination would only increase the observed disparity. Additionally, a peak in both the coercive field and the bias field for X = 0.27 is observed in Fig. 3d. This could be due to a balance between the disorder inherent to the entropy-stabilized system, which would increase the exchange bias, and a larger percent of magnetic ions, which would also be expected to increase the exchange bias^20,42,45^. As the mole fraction of Co is increased, the percentage of magnetic ions is greater, but the entropy of the structure decreases and thus an optimal Co concentration for maximizing the exchange coupling is expected.

In summary, we show that by utilizing the inherent chemical and structural disorder of entropy stabilized oxides, we can engineer a 10-fold increase in the exchange coupling with a ferromagnetic Py layer. Additionally, this phenomenon is strongly dependent on the concentration of Co in the sample, showing a relationship between chemical disorder and the density of uncompensated spins at the FM/AFM interface. Our results indicate that there are competing factors, degree of local disorder and concentration of magnetic ions, resulting in conditions that give rise to a maximum exchange coupling. As the concentration of Co in the samples is increased, we see an increase in the magnetic anisotropy along the [110] direction showing that, even with this large degree of disorder, entropy stabilized oxides can be intelligently engineered to take advantage of properties possessed by the constituent oxides while utilizing the inherent advantages of entropy stabilization.

## Methods

### Pulsed Laser Deposition

Targets were prepared by mixing and grinding the constituent binaries (MgO (Alfa Aesar, 99.99%), CoO (Alfa Aesar, 99.99%), NiO (Alfa Aesar, 99.99%), CuO (Alfa Aesar, 99.99%), and ZnO (Alfa Aesar, 99.99%)), then pressing the composite powder at 70,000 psi and sintering at 1100 °C for 18 hours in an air atmosphere. 80 nm thick films were deposited at 300 °C in 50 mtorr of O_2_ by ablation from a 248 nm KrF excimer laser fired at 6 Hz. A 2 nm Permalloy (Py) film was then deposited in vacuum at 40 °C and capped with 5 nm of Pt to prevent oxidation.

### X-Ray Diffraction

2*θ*-*ω* and reflectometry scans were performed on a Rigaku Smartlab diffractometer equipped with 1.54 Å Cu K*α* source and Ge-220 2-bounce monochromator. Reciprocal space maps (Sup. Figure 5) were conducted using the chi-phi goniometer and a second Ge-220 2-bounce monochromator on the acquisition side.

### Magnetometry

Magnetic properties of the exchange bias heterostructures were examined using a Quantum Design Dynacool 14 T Physical Property Measurement System. Samples were cooled from 350 K to 10 K under a 2 T field applied along the measurement axis. Isothermal magnetic hysteresis loops were then taken in 25 K increments back up to 350 K. Moment versus temperature curves were taken on a Quantum Design MPMS3 SQUID magnetometer by cooling the samples from 350 K to 5 K under 2 T (field cool) and 0 Oe (zero field cool), then measuring while warming to room temperature under a 50 Oe field.

### Atomic Force Microscopy

The topography of the samples was investigated using an NT-MDT NTEGRA Prima AFM in contact mode.

### X-ray Photoelectron Spectroscopy

XPS spectra were obtained using a Kratos Axis Ultra XPS with monochromated Al source and using a charge neutralizer. Spectra were taken from 1200 to 10 eV and high-resolution scans were taken about the Co, Ni, Cu, and Zn 2p peaks. These high-resolution scans were used for quantification.

## Electronic supplementary material

Supplementary Information
