# Supplementary material for: Giant Enhancement of Exchange Coupling in Entropy-Stabilized Oxide Heterostructures
Source: Sci Rep. 2017 Oct 17;7:13344. doi: 10.1038/s41598-017-13810-5 (PMC5645335; doi:10.1038/s41598-017-13810-5)
Supplement: Supplementary file 1 — Supplementary Information [file 41598_2017_13810_MOESM1_ESM.pdf]

Giant Enhancement of Exchange Coupling in Entropy-Stabilized Oxide Heterostructures

*P. B. Meisenheimer, T. J. Kratofil, J. T. Heron**

University of Michigan, Department of Materials Science and Engineering

2300 Hayward St, Ann Arbor, USA 48109

*Correspondence to: jtheron@umich.edu

Keywords: Entropy Stabilized, Oxides, Magnetism, Exchange Bias, Pulsed Laser Deposition

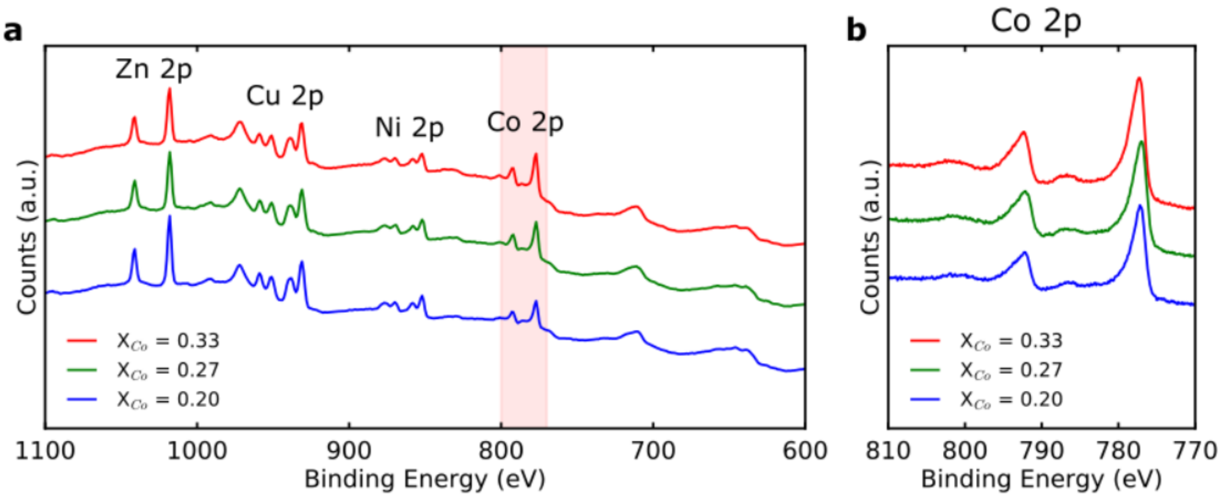

Element	X_{Co} = 0.20	X_{Co} = 0.27	X_{Co} = 0.33
**Co**	0.199 ± 0.026	0.273 ± 0.013	0.329 ± 0.027
**Ni**	0.196 ± 0.025	0.180 ± 0.008	0.170 ± 0.014
**Cu**	0.204 ± 0.032	0.180 ± 0.022	0.164 ± 0.033
**Zn**	0.201 ± 0.029	0.180 ± 0.013	0.162 ± 0.018

Supplementary Figure 1: X-ray Photoelectron Spectroscopy (XPS) of bare entropy-stabilized oxide films showing **a)** the full spectrum and **b)** a high-resolution scan about the Co 2p peak. The included table shows the quantification of the mole fractions from the XPS spectra above. The differences in concentration from the expected amounts are within the error of the scan resolution. The Co concentrations are highlighted for visibility. The shapes and positions of the XPS peaks are invariant for all the compositions in this study, indicating that there are no changes in the oxidation state as the composition is varied.

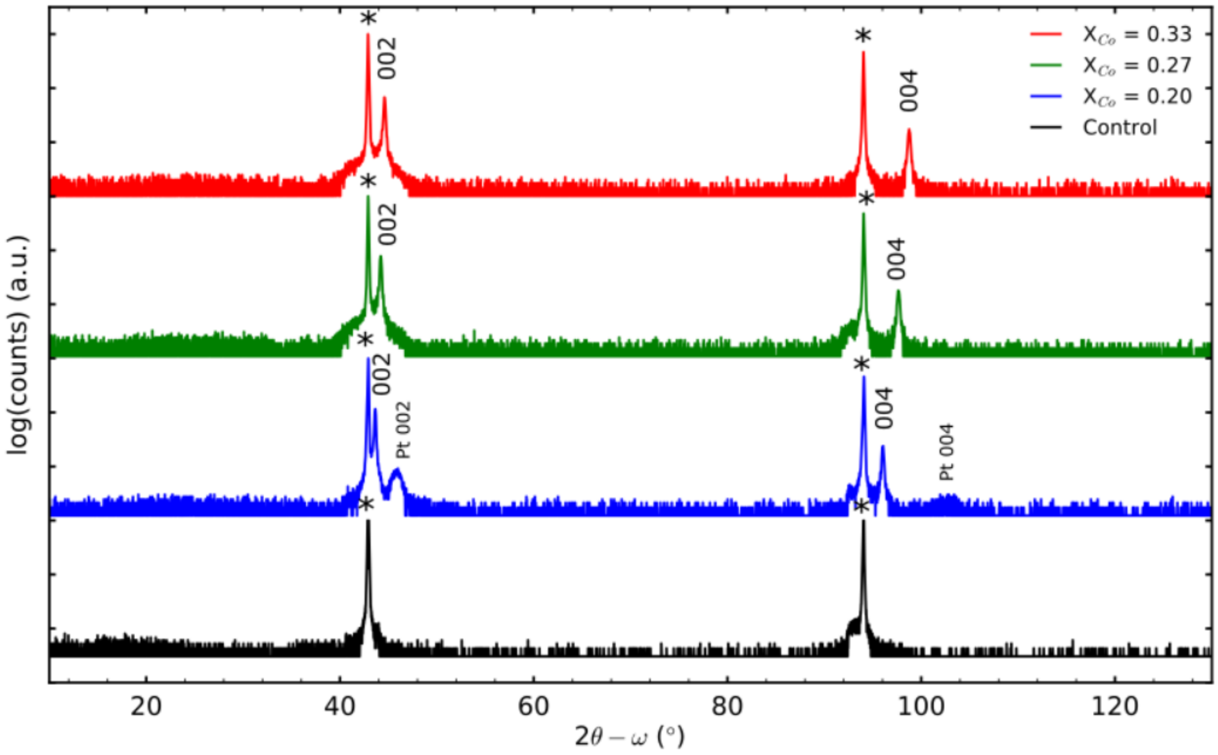

**X$_{Co}$**	c_0 **(Å)**
**0.20**	4.141 $\pm$ 0.0021
**0.27**	4.091 $\pm$ 0.0022
**0.33**	4.056 $\pm$ 0.0022

Supplementary Figure 2: Plot showing the full spectrum 2θ-ω X-ray diffraction patterns of the studied films, confirming the presence of no secondary phases. The X = 0.20 sample has a thicker (7 nm) Pt layer, explaining the small peak at ~45° and ~103°. The table shows the out-of-plane lattice constant values calculated using Cohen's method. The value c_0 is the corrected lattice constant from the equation: $\sin^2\theta = \frac{\lambda}{4}\left(\frac{h^2+k^2}{a_0^2} + \frac{l^2}{c_0^2}\right) + D\sin^2 2\theta$. Error bars correspond to the uncertainty in the peak position from the scan resolution.

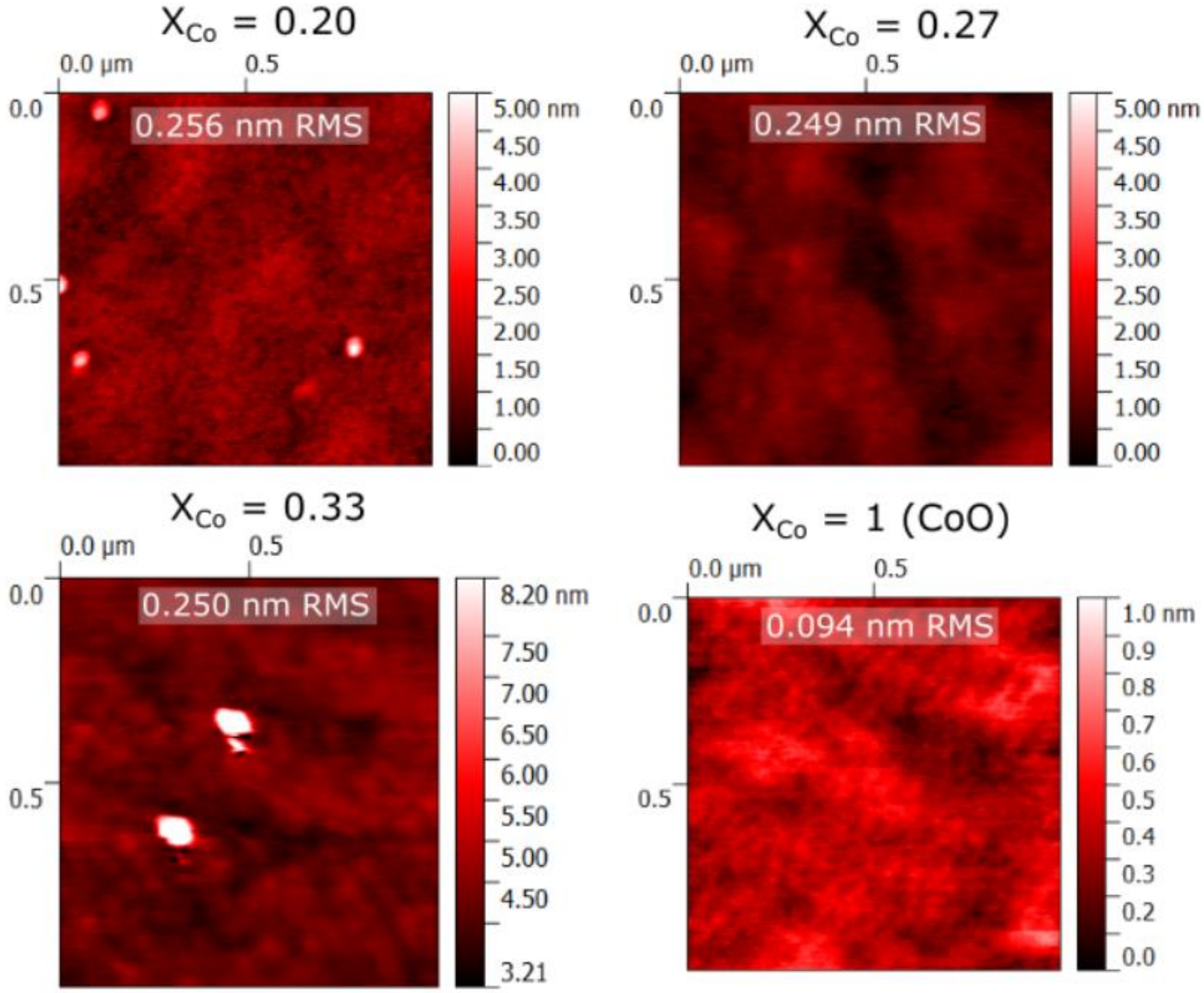

Supplementary Figure 3: Atomic force micrographs of bare (no Py) entropy-stabilized oxide and control films grown by PLD on single crystal MgO substrates. Images are tagged with root-mean-square roughnesses. For X = 0.20 and 0.33, the value shown is for in between the sparse particles. RMS for the entire image is 0.345 nm and 0.568 nm, respectively.

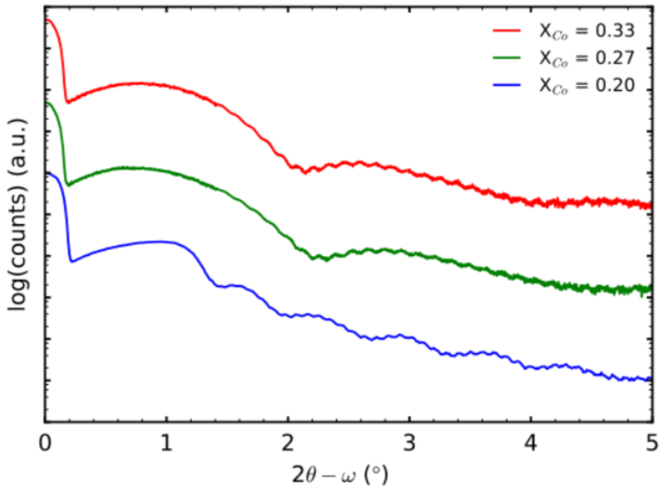

	Frequency (°)	d (nm)
**X_{Co} = 0.20**	0.113	78.0
**X_{Co} = 0.27**	0.118	74.8
**X_{Co} = 0.33**	0.11	80.2

Supplementary Figure 4: Low-angle X-ray reflectometry of exchange bias heterostructures. The period of the oscillations agrees well with the expected layer thicknesses, ~75-80 nm of oxide, 2.4 nm of Py, 4 nm of Pt. The X = 0.20 sample has a thicker (7 nm) Pt layer, resulting in the higher frequency oscillations in the blue curve.

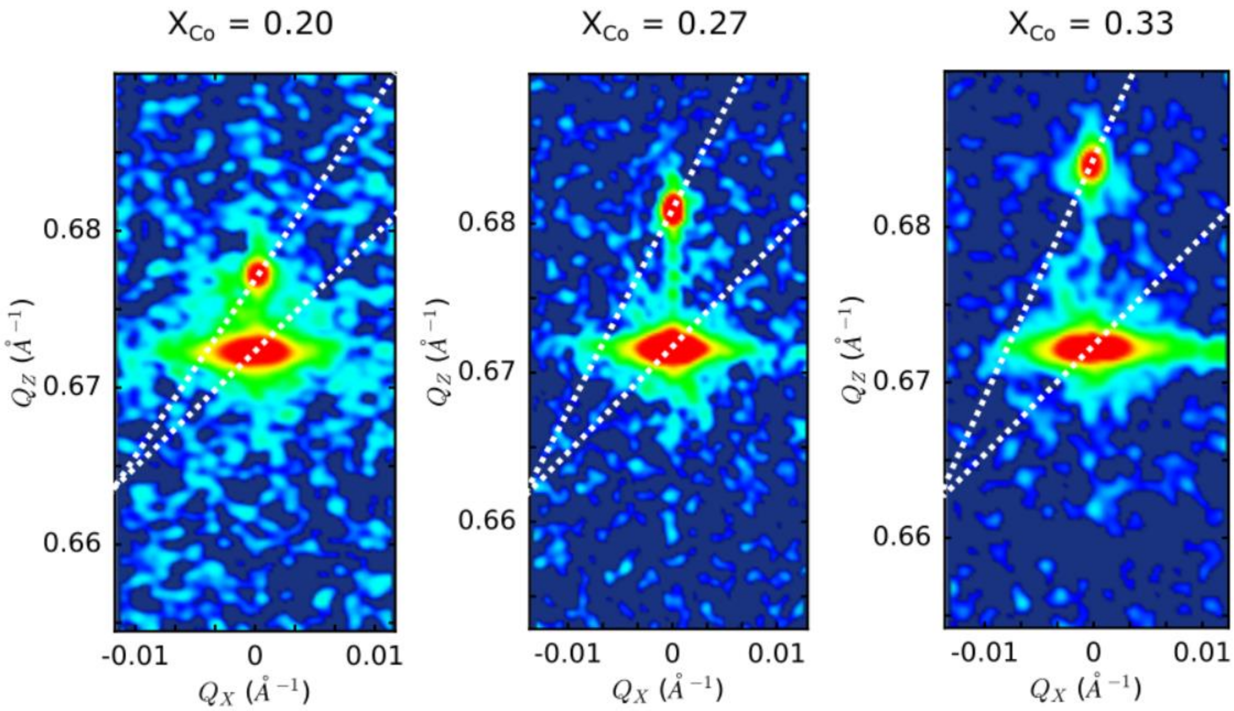

	c_0 (out-of-plane, Å)	a_0 (in-plane, Å)	**Difference (in-plane, %)**
**X_{Co} = 0.20**			
substrate	4.205 ± 0.0021	4.205 ± 0.0021	
film	4.141 ± 0.0021	4.205 ± 0.0021	0.011
**X_{Co} = 0.27**			
substrate	4.208 ± 0.0022	4.208 ± 0.0022	
film	4.091 ± 0.0022	4.207 ± 0.0022	0.0090
**X_{Co} = 0.33**			
substrate	4.207 ± 0.0022	4.207 ± 0.0022	
film	4.056 ± 0.0022	4.208 ± 0.0022	0.0091

Supplementary Figure 5: Reciprocal space maps of the film heterostructures about the 022 diffraction peak. The noncollinearity of the peaks with respect to the [202] reciprocal space vector (illustrated by the dashed lines) shows that the in-plane lattice constant of the film is

pinned by the substrate. Using the 2θ-ω values taken at $Q_X = 0$, the in-plane lattice constant of the film was determined using Cohen's method from the equation given in Sup. Figure 2. In all three cases, there is an approximately 0.01% difference between the substrate and film, agreeing with the observation that the peaks lie at the same Q_X position.

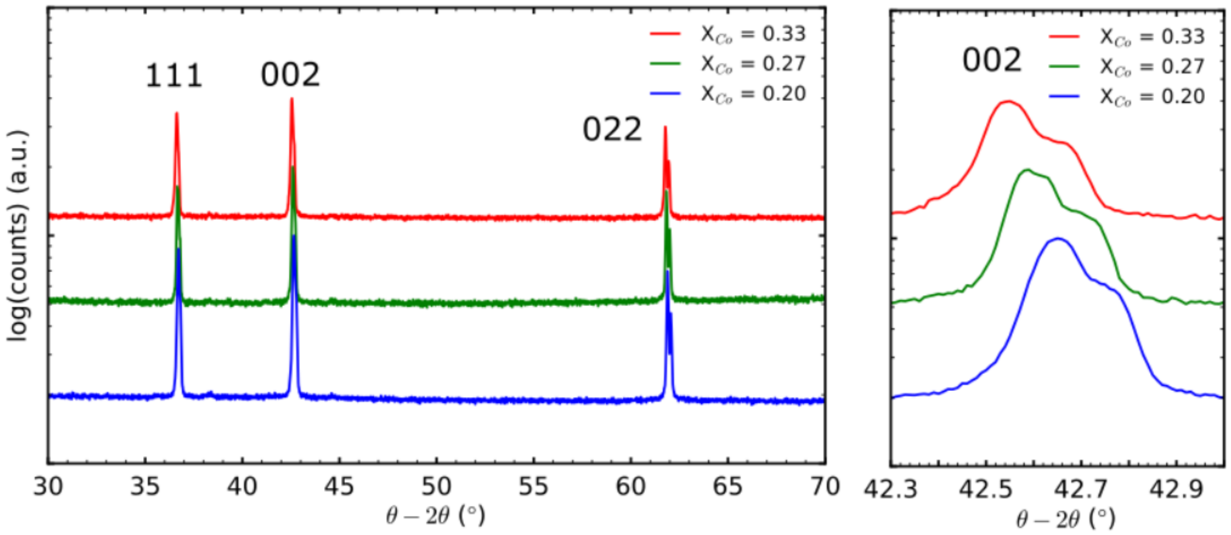

	a_0 (target, Å)	a_0 (film, Å)	ε
**X_{Co} = 0.20**	4.238 ± 0.00016	4.141 ± 0.0021	-0.022
**X_{Co} = 0.27**	4.248 ± 0.00072	4.091 ± 0.0021	-0.036
**X_{Co} = 0.33**	4.252 ± 0.00039	4.056± 0.0021	-0.045

Supplementary Figure 6: $\theta - 2\theta$ X-ray diffraction of the targets of varying composition in the range of the 111, 002, and 022 diffraction peaks. These curves were obtained on a diffractometer that has Cu Kα_1 and Cu Kα_2 radiation. Lattice parameters were determined from the Cu Kα_1 peaks using Cohen's method with the equation detailed above in Sup. Figure 2. Comparing the measured lattice parameters from the targets to those of the films, we retrieve our out-of-plane strain values of -2.2%, -3.6%, and -4.5%.

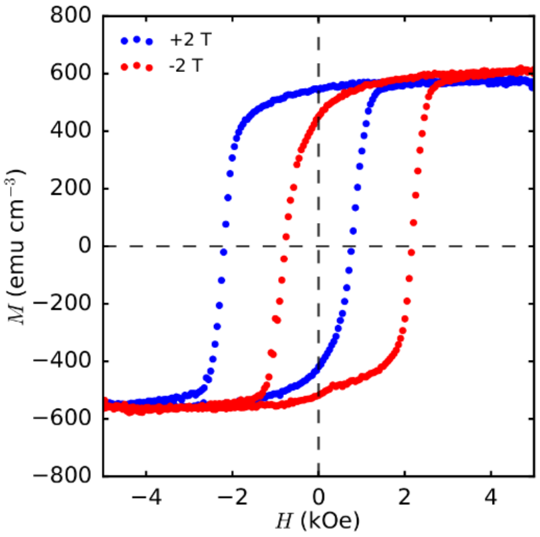

Supplementary Figure 7: Magnetic hysteresis loops for the X = 0.20 exchange bias heterostructure taken along the [100] direction at 10 K after field cooling in ± 2 T. The exchange bias is reversible upon reversal of the cooling field polarity.

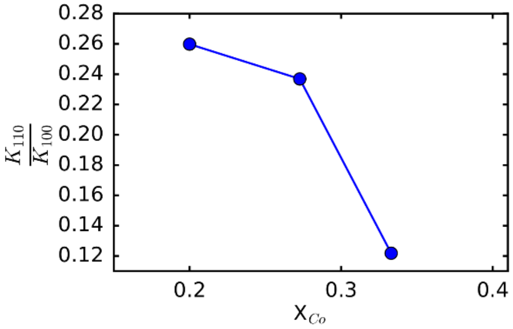

$\mathbf{X_{Co}}$	$\boldsymbol{K_{100}}$ **(J cm⁻³)**	$\boldsymbol{K_{110}}$ **(J cm⁻³)**	$\boldsymbol{\frac{K_{110}}{K_{100}}}$
**0.2**	0.01452	0.003773	0.259
**0.27**	0.1263	0.02991	0.237
**0.33**	0.03175	0.003868	0.122

Supplementary Figure 8: Plot showing relative anisotropy energies between the [110] and [100] crystallographic directions as a function of Co concentration. The anisotropy values were obtained by taking the integral $\int_0^{M_s} H(M)dM$ in the first quadrant of the hysteresis loop.

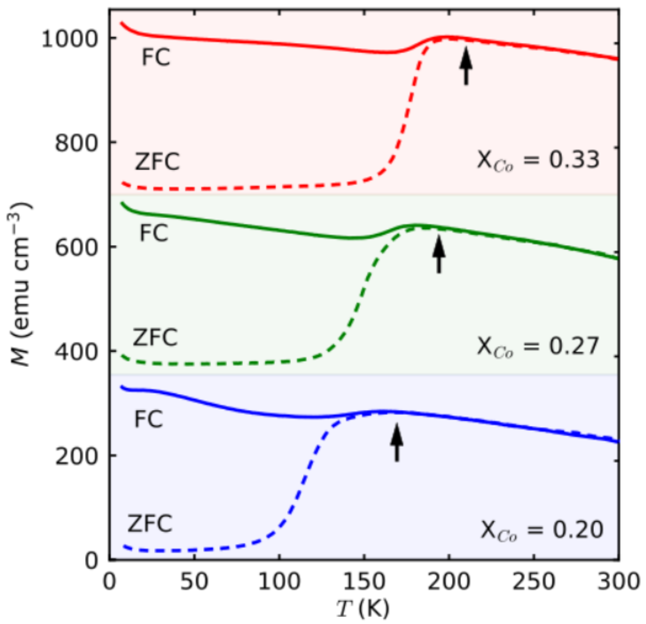

Supplementary Figure 9: Field cooled (FC) and zero field cooled (ZFC) moment versus temperature curves for the exchange bias samples. The curves are offset in y for clarity. We can directly extract the blocking temperature (T_B) of the samples from the temperature where the FC and ZFC curves differentiate beyond the noise floor, noted by the black arrows. The curves are very wide, spanning ~50 K, indicative of a sluggish phase change.
